# Parapagus Conjoined Twin : A Case Report

**DOI:** 10.31729/jnma.9180

**Published:** 2025-08-31

**Authors:** Kumar Bahadur Bista, Sharmila ghimire, Sandesh Acharya, Deepak Jung Subedi, Mamata Bista, Pramit Khatiwada

**Affiliations:** 1 Department of Gynecologic Oncology, Bhaktapur Cancer Hospital, Bhaktapur, Nepal; 2 Department of Radiology, Kathmandu Medical College Teaching Hospital and p.Ltd, Sinamangal, Kathmandu, Nepal; 3 Department of Obstetrics and Gynecology, Bheri Hospital, Banke, Nepal; 4 Department of Gynecologic Oncology, Bhaktapur Cancer Hospital, Bhaktapur, Nepal.; 5 Department of Anesthesia B.P Koirala Institute of Health Sciences, Dharal, Nepal; 6 Department of Gynecologic Oncology, Bhaktapur Cancer Hospital, Bhaktapur, Nepal

**Keywords:** *conjoined twins*, *twin pregnancy*, *thoraphagus*

## Abstract

This case was brought to our antenatal outpatient department at Bheri Hospital as a referral case from another center. She is a 23-year-old G3P1 at 20w2d gestation with one prior abortion. In her two Ultrasonographies done in a local clinic, no anomaly was detected, and she was referred for a routine anomaly scan through which conjoint parapagus twin was made. The treatment plan and outcome were discussed with the patient for a conjoined twin dicephalic parapagus type. Medical termination of pregnancy was performed, and she delivered a conjoined female of birth weight 1800 grams. This case illustrates the diagnostic and management challenges of dicephalus parapagus conjoined twins. Reporting such rare cases helps guide counseling, imaging, and individualized management in similar situations.

## INTRODUCTION

Twin pregnancy is rare, and conjoined twins are the rarest type of monochorionic twins. The incidence of conjoined twins among twin pregnancies is approximately 1 in every 200, and they are always identical. The overall incidence ranges from 1 in 50000 to 1 in 100000 live births.^1^ Although the exact cause is uncertain, it results from either a fission or fusion anomaly, resulting in incomplete embryonic division following 13 days of fertilization.^2^ Conjoined twins exist in a female-to-male ratio of 3 to 1. This type of pregnancy is high-risk, so it requires a coordinated multi-professional approach to manage it.^3^

## CASE REPORT

Mrs. S. Khan, a 23-year-old multigravida, was referred to the antenatal (ANC) out-patient department (OPD) of Bheri Hospital on 23 February 2025 at 20 weeks and 2 days of gestation by the last menstrual period. At that visit, regular prenatal examinations and an anomaly scan were performed. An anomaly scan revealed a dicephalic parapagus-type conjoined twin with two heads, one body, and two lower limbs. The placenta was posterior, and lab reports were within normal limits.

At the time of admission, the patient’s examination and vital signs were normal. She perceived good fetal movement, and her abdominal examination revealed a gestational age of 24-26 weeks with longitudinal lie. Confirmation was done again with a repeat scan, which revealed a 19 weeks and 6 days-old dicephalic twin at breech presentation with a posterior placenta and an adequate volume of amniotic fluid. After counseling regarding the diagnosis and poor prognosis, the parents and family decided to terminate the pregnancy.

**Figure 1:**
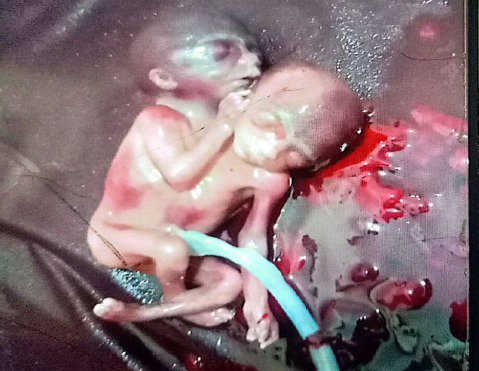
Conjoined twin.

**Figure 2:**
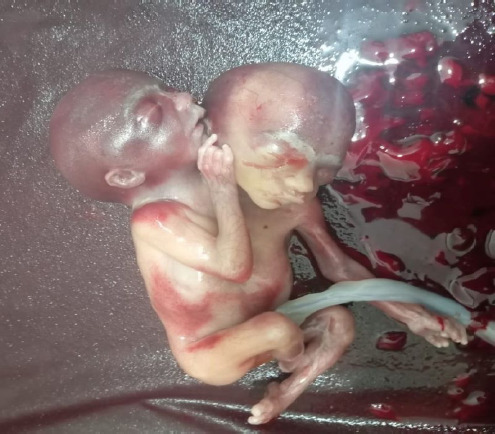
Conjoined twin.

After obtaining written consent, induction of labor with mifepristone followed by misoprostol was done. She delivered a conjoined dicephalus female fetus of birth weight of 1800 g, a placental weight of 300 g, and a foot length of 25cm on 27 February 2025 at 6:55 pm without any complication. A repeat scan on 28 February showed a retained product of conception measuring “36 mm × 34 mm × 29 mm” size for which misoprostol 600 microgram stat was given (Figure 1 and 2)

## DISCUSSION

Conjoined twins are also known as Siamese twins, named after the birthplace of the original Siamese twins, born in 1811 in Thailand.^4^ Conjoined twins are usually described by where they are joined, such as at the chest (thoracopagus) or abdomen (omphalopagus), with thoracopagus being the most common. Our case, however, involved the much rarer form known as dicephalic parapagus, where two heads share a single trunk and lower body. This subtype is associated with a particularly poor prognosis due to shared vital organs and high perinatal mortality.^5^ After a detailed examination and scan, in this case, we made a diagnosis of dicephalic parapagus of twin pregnancy as depicted in figure 1 and 2. Figure 1(Anterior view) and figure 2 (lateral view) shows parapagus conjoined twins, where the fetuses are fused side-by-side with a shared body axis. The thorax and abdomen appear fused, with a single umbilical cord visible in the image, suggesting a shared placenta and circulation This variant has high rates of antepartum, intrapartum, and postpartum complications. Imaging plays a vital role in the early identification of anatomical abnormalities. First-trimester dating scans facilitate early detection of significant anatomical anomalies. Routinely, all pregnancies should undergo a firsttrimester dating scan after 7 weeks of gestational age. In addition, the first-trimester anomaly screening scan should be done between 11-13 weeks of gestational age. Early detection of significant structural abnormalities and early intervention help to reduce the complications for the mother and family, both emotionally and physically.^5^ Conjoined twins are usually ruled out if two placentae or a membrane separating the twins are seen. Sonographic diagnostic criteria specific to conjoined twins are as follows: “no separating membrane, jointed body parts, and no separate body or head between the twins despite positional changes, bifid fetal pole in early scan and three-vessels cord”.^6^

The complexity of abnormalities on which prognosis depends is further explored with three-dimensional ultrasound, computed tomography, and magnetic resonance imaging after diagnosis of conjoined twins.^1^ Imaging, such as early prenatal ultrasound, fetal echocardiogram, and fetal MRI are useful modalities for diagnosis and to determine the prognosis of conjoined twins.^7^

In her two Ultrasonography done in local clinic, no anomaly was detected and she was referred for a routine anomaly scan through which diagnosis of conjoint parapagus twin was made. During the anomaly scan the conjoined twins with dicephalic parapagus-type was identified. Considering the complications and prognosis, termination with medical induction was done in this case. In the conjoined twins, the prognosis is guarded and associated with a high perinatal mortality. Therefore, parents and family members require detailed counseling regarding management options. Obstetricians are expected to provide thorough consultation to the parents in early gestational weeks and provide options for immediate termination of pregnancy.^8,9^

The management of conjoined twins is generally based on gestational age. In early gestation, medical induction of labor mostly leads to successful vaginal delivery; in one report 75% of cases were delivered vaginally whereas 18% underwent cesarean section in early gestation. A few cases with a period of gestation of 20 weeks underwent cesarean section. Careful induction of labor should be done after 20 weeks of gestation in conjoined twins. As gestational age advances, likelihood of cesarean section increases.^10^

The conjoined twins represent a rare clinical condition that is encountered during our daily case management. Therefore, for health professionals directly involved in this sort of case management, a basic understanding of the management plan and difficulties related to this illness is required.

## Consents:

JNMA Case Report Consent Form was signed by the patient and the original article is attached with the patient’s chart.
